# Articulating Artificial Teeth

**Published:** 1880-08

**Authors:** W. E. Driscoll


### ARTICULATING ARTIFICIAL TEETH.

*Read before the Indiana State Dental Society.*

BY W. E. DRISCOLL.

Mr. President:—I wish to call attention to a plan for retaining artificial teeth in position where the gums are short. The same idea may have been given the profession before, but I have never seen anything of it in our literature, or heard a word from any one on the subject. If any one has ever heard the plan proposed before, I will be glad if he will communicate the fact to me. I was led to adopt the plan by having observed that a large proportion of bad fits were caused by the upper teeth being pressed outward by some fault in their construction. Hence, any arrangement that would press an upper set backward and a lower set forward, I reasoned, would be a great assistance in their retention in position. Where the inferior natural bicuspids are retained and it is a full upper set that is to be made, the backward pressure may be secured by allowing an artificial bicuspid or molar to project obliquely behind the last natural tooth on each side, in such manner as to cause a glancing stroke against the projecting anterior surface of the artificial bicuspid or molar that will press it back at every closure of the jaws.

Where there are no natural bicuspids or first molars, this backward pressure of an upper set must be secured by sinking the inferior molars as low on the gums as can possibly be done even with teeth constructed short from the pins to the grinding surfaces. From the posterior molar forward the ascent on the grinding surfaces must be as great as the superior bicuspids will permit, when they also are short from the pins to their grinding surfaces and are set as far upward as possible. The cuspids and incisors are to be set so that the upper and lower ones will not strike each other as hereafter described, but *may* be made to show in their proper proportions even though there may be necessity for the superior cuspids to be considerably longer than the bicuspids posterior to them. Of course this contemplates making the superior molars exceedingly long in cases where there is a great deal of space between the gums. Still it is the proper thing to do even if the grinding surfaces of the superior molars were one inch from the upper gum and the grinding surfaces of the inferior molars were no more than one-eighth of an inch from the lower gum.

When a new set of teeth are first inserted, the molars should come firmly together, but no teeth anterior to them should be able to catch and hold a strip of muslin when the jaws are closed as firmly as possible,

When the bite is taken, if the operator will place a finger on the middle of each masseter muscle and direct the patient to close the jaws like he would if aiming to crush something in that part of the mouth on both sides at once, the patient will then as a rule close the lower jaw about one-eighth of an inch back of the position that will generally be occupied by it in relation to the superior maxilla.

This being true, and the only way I know of, in which we can depend upon the bite given by the patient, we must in setting up the teeth, make allowance for a shifting of the lower jaw forward at least one-eighth of an inch. If we do not, and the superior incisors lap closely in front of the inferior incisors then the upper plate will be pushed forward at every closure of the jaws, which is the result I am particularly aiming to show a way to avoid.

When all or nearly all the inferior natural teeth are retained, we sometimes find the second or third inferior molars very much elevated above a plane with the bicuspids and incisors. So much so, in some cases that the molars if they strike hard against a full upper set, have the effect of pressing the upper set forward and a bad fit is the consequence although the upper gums may be first-class for retaining a plate. When this is the case the only teeth that must be allowed to approach closely enough to hold a strip of muslin, are the bicuspids, or if there is a molar that is not elevated above a level with the bicuspids then it may also be allowed to come in firm contact with the upper teeth.

The portion of the plate that extends back of the teeth on it, may be allowed to rest upon the apex of the last inferior molar in such a way as to not receive any forward propulsion and still assist in balancing a plate in a difficult case. Quite often this will keep a plate from dropping down when nothing else will secure it near so well. It is a good plan to allow the imprint of the last molar to remain in the wax plate until the case is placed in the mouth, and when necessary the plate may be thickened somewhat to meet the tooth it is to rest upon.

A large proportion of the full sets of artificial teeth, we see, have the upper anterior teeth extending downward so much that we detect their artificial character at a glance. It would seem that many in the business of making artificial teeth, have not noticed that this may in a great measure be overcome by the proper selection of the teeth, that is using those very short from the pins to the cutting edges of the teeth. I mention this in this connection as it is often necessary to use such teeth to give them the necessary "dip" from the anterior to the posterior tooth.

I wish to say in conclusion, that I realize that it is not an easy matter to find anything that will be entirely new to every one who may hear this description. If we abstained from contributing our mites to the general fund of information, from a fear of some one thinking he could have told it better, then our progress will be slow indeed. I also recognize the fact, that it will be difficult for many to understand exactly what I am aiming to explain in all parts of this paper, but I hope all will get a general working idea, and if they do, and put the suggestions to the test of practice, I would like to hear from each one what he thinks of the plan, as well as any suggestion for improvements upon it that may be worked out by them.

